# Development and Evaluation of an AI-Assisted Answer Assessment (4A) for Cognitive Assessments in Nursing Education

**DOI:** 10.3390/nursrep15030080

**Published:** 2025-02-26

**Authors:** Piyanut Xuto, Piyaporn Prasitwattanaseree, Tareewan Chaiboonruang, Sujitra Chaiwuth, Podjanee Khwanngern, Chadchadaporn Nuntakwang, Karnjana Nimarangkul, Wara Suwansin, Lawitra Khiaokham, Daniel Bressington

**Affiliations:** 1 Faculty of Nursing, Chiang Mai University, Chiang Mai 50200, Thailand; piyaporn.p@cmu.ac.th (P.P.); tareewan.c@cmu.ac.th (T.C.); sujitra.c@cmu.ac.th (S.C.); podjanee.p@cmu.ac.th (P.K.); chadchadaporn.n@cmu.ac.th (C.N.); karnjana.chai@cmu.ac.th (K.N.); prueksalada.k@cmu.ac.th (L.K.); 2 Srisavarindhira Thai Red Cross Institute, Bangkok 10330, Thailand; wara.s@stin.ac.th

**Keywords:** AI-assisted assessment, nursing education, cognitive assessment, educational technology

## Abstract

Artificial intelligence (AI) can potentially enhance cognitive assessment practices in maternal and child health nursing education. **Objectives**: To evaluate the reliability, accuracy and precision, and external validity of an AI-assisted answer assessment (4A) program for cognitive assessments in nursing education. **Methods**: This study is a validation study. Initially, 170 nursing students from northern Thailand participated, with 52 randomly selected for detailed testing. Agreement testing between the 4A program and human experts was conducted using the intraclass correlation coefficient (ICC). Accuracy and precision testing compared 4A scores with human expert assessments via the McNemar test. External validation involved 138 participants to compare the 4A program’s assessments against national examination outcomes using logistic regression. **Results**: Results indicated a high level of consistency between the 4A program and human experts (ICC = 0.886). With an accuracy of 0.808 and a precision of 0.913, compared to the human expert’s accuracy of 0.923 and precision of 1.000. The McNemar test (χ^2^ = 0.4, *p* = 0.527) showed no significant difference in evaluation performance between AI and human experts. Higher scores on the 4A program significantly predicted success in the national nursing examination (OR: 1.124, *p* = 0.031). **Conclusions**: The 4A program demonstrates potential in reliably assessing nursing students’ cognitive abilities and predicting exam success. This study advocates for the continued integration of AI in educational assessments and the importance of refining AI systems to better align with traditional assessment methods.

## 1. Introduction

It is essential to evaluate nursing students’ cognitive abilities, as critical thinking is required to deliver high-quality patient care and make evidence-based decisions. Artificial intelligence (AI)-assisted evaluation approaches have the potential to supplement human assessments and assignment marking in the quickly changing field of healthcare education [1]. According to Swiecki et al. [2], it is well established that traditional assessments, such as multiple-choice questions and paper-based exams, have limitations in capturing the dynamic nature of nursing practice and evaluating the cognitive competencies required in modern nursing. This is particularly pertinent for some digital-based learning tasks. Consequently, there is an increasing need for creative evaluation techniques that meet the needs of the digital age.

### Background

Cognitive capabilities in nursing students are integral to their ability to provide safe patient care and make informed decisions in a complex healthcare environment [3]. These capabilities encompass a range of critical processes for effective clinical and moral reasoning, critical thinking, decision-making, and situational awareness [4]. Effective clinical and moral reasoning allows nursing students to navigate inpatient care’s ethical and clinical complexities [5]. They must be able to understand and balance ethical principles with clinical evidence, making decisions that are not only medically sound, but also ethically responsible. This involves a deep understanding of patient rights, dignity, and cultural considerations and the ability to apply clinical knowledge to real-world situations [6]. Critical thinking is central to nursing practice, as it requires analyzing and synthesizing information, evaluating evidence, and challenging assumptions. It involves self-regulated, purposeful skills that enable nurses to adapt to complex clinical scenarios through logical reasoning and contextual understanding [7]. Nursing students utilize critical thinking to interpret patient data, recognize patterns, and identify potential issues before they escalate into problems. The new digital teaching approaches, such as simulation-based learning, significantly enhance critical thinking by engaging students in reflective practices that promote pattern recognition and proactive problem-solving [8].

A recent systematic review found that technology-supported critical thinking models provide learning strategies via demonstration, discussion, and reflection [7]. This skill is crucial in assessing patient needs, planning care, and evaluating outcomes. Decision-making in nursing is often a complex process that requires quick thinking and sound judgment. Nursing students must weigh various factors, including patient conditions, treatment options, and resource availability. Effective clinical decision-making integrates theoretical and practical knowledge, enabling students to critically evaluate patient conditions, identify priorities, and implement evidence-based interventions to make the best possible decisions for patient care [9]. Situation awareness refers to the gathering and processing of relevant information to know what is going on and anticipate what might happen shortly [10]. In a healthcare setting, this means being cognizant of the dynamics of the patient’s condition, the functioning of the healthcare team, and the availability of resources.

In light of contemporary schooling and technological improvements, online cognitive exams have become a more suitable substitute for conventional multiple-choice questions [11]. They give nursing students the option to take exams whenever it is most convenient for them, which can be very helpful for those who are juggling other obligations or competing schedules. Students from various regions can participate without having to physically be present, because this flexibility also transcends geographic borders. The integration of technology in online examinations enables a broader range of question formats, including interactive scenarios, simulations, virtual labs, and multimedia content, enhancing student engagement and learning experiences [12]. Compared to typical multiple-choice examinations, this variation can make assessments more engaging for students and more closely resemble real-world clinical settings [13]. The actual application of information is just as crucial in nursing education as in academic theory and understanding. A more accurate indicator of a student’s capacity to apply knowledge in practical contexts can be found in these interactive formats. Educational technology plays a crucial role in modern nursing education by enhancing student engagement, facilitating personalized learning, and improving assessment methodologies. Traditional assessment tools, such as multiple-choice exams, have limitations in evaluating complex cognitive skills. The adoption of AI in educational technology offers opportunities for more dynamic and adaptive assessment approaches, ensuring better alignment with contemporary nursing competencies.

Artificial intelligence is increasingly being integrated into nursing education, particularly in grading assignments and assessments. AI-powered grading tools, such as those embedded in learning management systems like Mango Canvas, primarily focus on structured assessments, including multiple-choice questions and automated scoring of written responses based on predefined rubrics [1,2]. However, these systems often lack the ability to assess nuanced cognitive skills, such as critical thinking, clinical reasoning, and decision-making, which are essential in nursing practice [13,14]. The 4A program differs from conventional AI grading tools by employing speech-to-text (STT) and natural language processing (NLP) to assess students’ verbal reasoning in cognitive assessments. By analyzing open-ended audio responses, the 4A program provides a more comprehensive evaluation of cognitive abilities, offering a validated alternative that aligns with human expert judgment [15].

This study introduces the 4A system as an advanced AI solution aiming to provide a more comprehensive cognitive assessment tool for nursing education. This research aims to develop and evaluate the 4A program by comparing three types of innovation testing: (1) agreement testing to ensure consistency between the AI system and human experts [16], (2) accuracy and precision testing to measure the system’s ability to classify responses correctly and without bias [17], and (3) external validation to confirm the generalizability and real-world applicability of the program [17].

## 2. Materials and Methods

This study is a validation study aimed at evaluating the 4A program, an AI-driven cognitive assessment tool for nursing education. The validation process employs cross-sectional data and statistical methods to establish the tool’s reliability, accuracy, and generalizability in educational contexts. The research was conducted in two main phases: initial validation and external validation. Each phase was designed to rigorously assess different capabilities of the 4A program through agreement testing, accuracy testing, and external validation.

### 2.1. Study Sample and Setting

The sample of fourth-year nursing students was recruited from a nursing education institution in Northern Thailand. The institution predominantly provides an undergraduate educational program for around 960 nursing students. The sample size consisted of 308 participants, divided into two rounds, the first round included 170 nursing students, whereas the second round had 138 participants.

#### 2.1.1. Inclusion Criteria

Participants were eligible for this study if they met the following criteria: (1). Academic Standing: Individuals must be fourth-year nursing students who have completed all required courses in maternal and child health nursing. (2). Examination Preparation: Students must be actively preparing for the national nursing examination certificate as administered by the Thailand Nursing and Midwifery Council. (3). Geographical Location: Participants must be enrolled in a nursing educational institute located in northern Thailand. (4). Technological Requirements: Students should have access to a functional internet-connected device equipped with a microphone that can record sound, ensuring they can engage effectively with the 4A program for online cognitive skills assessments.

#### 2.1.2. Exclusion Criteria

Participants were excluded from the study under the following circumstances: (1). Electrical Shutdown: Individuals experiencing an electrical power outage during the testing session, preventing the completion of the assessment. (2). Internet Connectivity Issues: Participants who lose internet connectivity during the testing period, inhibiting their ability to effectively participate in the online assessment.

#### 2.1.3. Sample Size: Initial Validation Phase

The sample size for the initial validation phase, encompassing both agreement and accuracy testing, was determined using power analysis. The parameters for the power analysis included the choice of a moderate effect size of 0.5. This effect size aligns with findings from prior work using virtual nurse labs, where similar outcomes were observed [18]. Power (1 − β) 0.80, and significance level (α) 0.05 were used. Using G*Power 3.1.9.4 software, these parameters indicated a required sample size of 52 participants for robust testing of agreement and accuracy. This sample size ensures the reliability of comparisons between the assessments made by the 4A program and human experts, as well as the evaluation of the 4A program’s accuracy and precision.

#### 2.1.4. Sample Size: External Validation Phase

For the external validation phase, the 4A system’s outputs were compared with the national nursing examination results. Using the rule of thumb for logistic regression, at least 20 events per variable are required for a robust model. Using the average pass rate of 82.29% (failure rate 17.71%) from the Nursing and Midwifery Council’s national examination data over the past three years (personal communication in December 2022). The required sample size was calculated as 20/0.177 = 113 participants. Accounting for a 20% attrition rate, the total number of participants needed was approximately 138.

### 2.2. Instrumentation

The research instruments utilized in this study consist of two primary components designed to align with the learning outcomes of the nursing curriculum, specifically targeting maternal and child health nursing practice: (1) Scenario-Based Assessments: A series of three detailed case studies requiring audio responses from participants in terms of nursing diagnosis, signs or symptoms, and nursing care. These scenarios were crafted to evaluate the students’ critical thinking, problem-solving, and decision-making skills in maternal and child health contexts. Each scenario prompted the student to articulate responses that reflect their clinical reasoning and ability to handle complex healthcare situations effectively. The maximum possible summed score for the three scenarios was 38 points. (2) Data Collection Questionnaires: These instruments were composed of two parts: (2.1) Personal Information: gender, hometown, primary language used, main device used for testing, and grade point average, (2.2) Perception of Cognitive Skill Assessment via AI. This questionnaire was developed to assess participants’ responses to an AI-assisted answering assessment program, focusing on their perception of cognitive abilities in decision-making, particularly when these abilities are enhanced or evaluated through an AI system. It consists of 10 items, measured on a 5-point Likert scale, with each item rated from 1 (strongly disagree) to 5 (strongly agree), resulting in a total possible score of 50.

Interpretation of Scores:

- 41–50: Highly positive perception—Participants strongly agree that the AI system effectively enhances and evaluates their cognitive skills.
- 31–40: Positive perception—Participants agree that the AI system positively contributes to their cognitive skills, though there may be minor reservations.
- 21–30: Neutral perception—Participants have mixed feelings about the AI system’s impact on their cognitive skills.
- 11–20: Negative perception—Participants disagree that the AI system significantly enhances or evaluates their cognitive skills.
- 1–10: Highly negative perception—Participants strongly disagree and perceive the AI system as ineffective in supporting their cognitive skills.

### 2.3. Validation and Reliability

The 4A program underwent both alpha and beta testing phases. During the alpha testing phase, research staff conducted tests in controlled environments to detect and address technical issues before exposing the program to users. The beta testing phase was extended to a small group of 15 recently graduated nursing students to evaluate the usability and performance in real-world scenarios. Feedback gathered during this phase was used to optimize the program further, ensuring that the final version of the 4A program met user expectations and operated effectively. The research instruments, such as scenario-based assessments and perception of cognitive skill assessment via AI, were rigorously reviewed and validated by a panel of six experts in the field, including two in nursing education, two specializing in obstetric nursing, one midwifery expert, and one expert in AI-generated content. The content validity index (CVI) for the scenario-based assessments and perception of cognitive skill assessment via 4A were calculated to be 0.98, and 0.98, respectively. The perception of cognitive skill assessment via 4A was evaluated for internal consistency using Cronbach’s alpha, which yielded a value of 0.96.

### 2.4. Data Collection

Data collection was conducted in two distinct phases to validate the 4A program and evaluate its efficacy in a real-world educational setting. Phase One (2022) was the initial validation. A total of 170 nursing students participated in this phase. Each participant completed an online cognitive assessment to test cognitive abilities in scenarios relevant to maternal and child health. Participants were allocated 15 minutes per scenario, with three scenarios totaling 45 minutes. The audio responses collected were utilized to train the 4A program, enhancing its capability to convert spoken words into written text accurately. The transcribed texts were analyzed using natural language processing (NLP) techniques against a set of standard answers to evaluate agreement and accuracy in the assessment responses. The 4A program in this study utilizes a combination of computer algorithms and artificial intelligence (AI) to evaluate students’ audio responses in online cognitive skill assessments. The text analysis component of this program is powered by ChatGPT-4o API, a natural language processing (NLP) model designed to process and evaluate textual data. The process involves two main steps: (1) speech-to-text conversion (STT), the program converts spoken responses into written text using automatic speech recognition (ASR). A dataset of 170 transcribed audio responses was used to fine-tune the accuracy of the model, ensuring its effectiveness in recognizing technical nursing vocabulary, patient scenarios, and procedural descriptions. And for (2) text analysis via NLP, once transcribed, student responses undergo semantic similarity analysis using ChatGPT-4o API, which compares each response to predefined model answers. Responses with an overlap score of ≥60% are considered acceptable. This approach enhances objectivity in assessment by leveraging ChatGPT-4o’s contextual understanding and pattern recognition capabilities, aligning with recent advancements in AI-driven educational technologies [19].

### 2.5. Agreement Between 4A Program and Human Experts

The agreement testing involves comparing the 4A program’s output against the assessment of two human experts. This helps to evaluate the reliability of the 4A program assessment compared to human judgment. A subset of 52 participants was randomly selected from the 170 participants using the ‘Select Cases’ random sampling feature in SPSS (version 18). Audio clips from all three scenarios for these 52 participants were gathered during the initial testing phase. Two independent experts (referred to as CN and KN) were assigned to listen to and evaluate the selected clips. Each expert independently verified the responses, assessing their accuracy and relevance to the scenarios posed. To measure agreement, the evaluations provided by the 4A program for these clips were compared with the independent evaluations of the two human experts. This comparison aimed to determine the level of concordance among the three assessment sources, focusing on whether the program’s responses aligned with expert judgments.

### 2.6. Accuracy and Precision of the 4A Program

Accuracy refers to how well the 4A program correctly identifies both correct and incorrect responses, providing an overall measure of correctness. Precision, on the other hand, focuses on how well the 4A program identifies correct answers without mistakenly labeling incorrect answers as correct. In simpler terms, accuracy is about overall correctness, while precision is about avoiding false positives [2,20]. Using the same set of 52 clips, the accuracy and precision of the 4A program were assessed based on its classifications compared to those made by the human experts. Accuracy evaluates the overall correctness of the 4A program’s classifications. It is defined as the proportion of correct classifications (both true positives and true negatives) relative to all classifications made. In this context, accuracy measures how effectively the 4A program distinguishes correctly from incorrect responses provided by students. Precision focuses on the program’s reliability in identifying correct responses. It is defined as the proportion of true positives (correct answers identified by the program) out of all instances classified as correct by the program. Precision assesses the program’s ability to avoid misclassifying incorrect responses as correct.

### 2.7. External Validation

Phase Two (2023) involved an external validation process. The primary aim was to externally validate the 4A program by comparing its online cognitive test results with the outcomes of the national nursing licensure examination. The external validation was to confirm the findings from the beta testing and ensure the program’s effectiveness and generalizability across different groups of users to measure cognitive ability. In this phase, participants were openly invited to take the test as part of their preparation for the national licensure examination. A total of 138 participants from three nursing institutions in the northern part of Thailand participated in this phase. Each participant completed the online cognitive test and later reported their pass or fail results from the national nursing licensure examination. The outcomes from the 4A program were then compared with the results of their national licensure examination to evaluate the program’s generalizability and predictive validity within a broader educational context.

### 2.8. Data Analysis

Initial analysis involved computing descriptive statistics to summarize the demographic characteristics of participants. Means, standard deviations, and frequencies were calculated to provide a comprehensive overview of the study population using SPSS version 18. The intraclass correlation coefficient (ICC) was utilized to assess the consistency of verification methods across the different sources of answers: two human experts and the human expert with the 4A program. Accuracy and precision evaluation were used via the calculation of true positives, true negatives, false positives, and the total number of cases. The accuracy is calculated from (true positives plus true negatives) divided by total number of cases. Whereas precision is calculated from true positives divided by (true positives plus false positives). McNemar’s test was used to compare the accuracy and precision between 4A and human experts with a significance level set at *p* < 0.05. Logistic regression was employed to analyze the capability of the 4A program’s assessments in predicting students’ success on the national nursing examination.

### 2.9. Ethical Considerations

This study received prior approval from the Institutional Review Board, ensuring compliance with ethical standards for research involving human participants. All participants were provided with detailed informed consent forms before their participation. These documents clearly explained the study’s purposes, procedures, potential risks, and benefits. Participants were assured that they had the right to withdraw from the study at any point without any consequences, ensuring voluntary participation. Strict measures were implemented to maintain the confidentiality and anonymity of participant data throughout the study. Personal identifiers were removed from all study documents and datasets to prevent any breach of privacy. Protocols for data handling and security were rigorously followed. These included secure storage of digital data and restricted access to ensure that participant information was protected against unauthorized access, use, disclosure, disruption, modification, or destruction. Participants were kept informed about the progress of the study and their rights as research subjects. The study adhered to ethical considerations to ensure participant rights and safety, receiving approval from the Institutional Review Board of the Faculty of Nursing at Chiang Mai University (Study Protocol Code: 2023-EXP003). The research protocol underwent an expedited review process and received official ethical approval on 13 February 2023 (approval valid until 12 February 2024).

## 3. Results

From Table 1, the sample size consisted of 308 participants, divided into two rounds, the first round included 170 nursing students, whereas the second round had 138 participants. The vast majority of participants were female. Most participants were from the North region, representing 81.76% in Round 1 and 81.85% in Round 2. The primary language used by the majority was “Central” Thai, with 55.88% in Round 1 and 65.94% in Round 2. The iPad was the most used device in both rounds, accounting for 59.41% in Round 1 and increasing to 64.49% in Round 2. Notebooks were the second most used device, while smartphones and PCs saw less usage across both rounds. The mean GPA of participants was similar between rounds, at 3.08 (SD = 1.28) in Round 1 and 3.14 (SD = 1.32) in Round 2. The proportion of participants who did not pass the AI assessment was high in both rounds: 68.82% in Round 1 and 69.57% in Round 2. Data from Round 2 showed that 88.41% of participants passed the national examination.

### 3.1. Agreement Between 4A Program and Human Experts

From Table 2, the agreement testing between the AI-assisted answer assessment (4A) program and human experts yielded high consistency. The intraclass correlation coefficient (ICC) for the agreement between the two human experts was 0.971 (95% CI: 0.832–0.995, *p* = 0.001), indicating excellent reliability. When comparing the AI system with the human experts, the ICC was 0.886 (95% CI: 0.334–0.980, *p* = 0.009), which also reflects strong agreement, though slightly lower than the inter-expert agreement. These findings suggest that the 4A system is highly consistent with human judgment in evaluating cognitive assessments.

### 3.2. Accuracy and Precision of the 4A Program

The performance of the 4A program and human experts in terms of accuracy and precision were tested. The results show that the 4A program achieved an accuracy of 0.808 and a precision of 0.913, while the human expert demonstrated higher accuracy (0.923) and perfect precision (1.000). The McNemar test for agreement yielded a χ^2^ value of 0.4 (*p* = 0.527), indicating no statistically significant difference between the 4A program and the human expert in their evaluation performance. Please see Table 3 for results.

### 3.3. External Validation

The external validation phase involved comparing 138 students’ 4A program scores with their results (pass/fail) on the national nursing examination. The logistic regression analysis revealed that higher scores on the 4A system significantly predicted success in the national examination, with an odds ratio of 1.124 (95% CI: 1.012–1.245, *p* = 0.031), indicating that for every one-point increase in the 4A program score, the odds of successfully passing the national nursing examination increased by 12.4%. This result confirms the predictive validity of the AI system, suggesting that students who perform well on the 4A assessment are more likely to succeed in national certification exams. Please see Table 4 for results.

From Table 5, participant feedback on the 4A program was generally positive. The overall rating for the interest in online cognitive skill measurement was high, with a mean score of 4.29 (SD = 0.78). Participants also indicated that the assessments effectively reflected their cognitive skills (mean = 4.35, SD = 0.79) and that the questions were clear (mean = 4.17, SD = 0.87). The scenarios were perceived as stimulating cognitive skills (mean = 4.49, SD = 0.71), and the majority of participants felt that the assessment could be applied to other competency areas (mean = 4.34, SD = 0.85). However, the feedback also indicated that while voice-recorded responses were valued, their ability to measure cognitive skills better than multiple-choice questions was somewhat less agreed upon, with a mean score of 4.10 (SD = 1.05).

## 4. Discussion

The first objective of this study was to evaluate the level of agreement between the 4A program and human experts in assessing nursing students’ cognitive skills when faced with the three learning scenarios. The findings revealed a high level of consistency between the 4A program and human evaluators, with an intraclass correlation coefficient (ICC) of 0.971 between the two human experts and 0.886 between the AI program and human experts. These results indicate that the 4A program is highly reliable and closely aligns with expert judgment, suggesting its potential as a dependable tool in educational assessments. The program’s ability to replicate human performance is supported by findings that structured testing, whether conducted by AI or human evaluators, enhances cognitive skills through retrieval practices and reinforcement [21,22]. The high ICC value between the two human experts supports the reliability of expert assessment methods. This is consistent with findings from previous studies that emphasize the importance of reliability in educational assessments, particularly in high-stakes testing environments where consistency is critical to ensuring fairness and accuracy [23].

However, the slightly lower ICC between the AI program and the human experts, though still strong, reflects the inherent challenges of algorithmic assessment. Unlike human assessors, 4A systems rely on predefined criteria and algorithms that may not capture the full complexity of cognitive assessments, such as nuanced understanding and contextual interpretation. This limitation is echoed in the literature, where studies have highlighted the difficulty of developing an AI program that can fully replicate human judgment in complex tasks [2]. Another potential reason for the lower agreement between the AI system and human experts could be attributed to the primary language used by participants, particularly those from the northern region of the country. A significant proportion of participants spoke a regional dialect as their main language, which may have introduced challenges in accurately interpreting responses during the AI assessment. Unlike human experts, who can adapt to linguistic nuances and contextual variations, the AI system may struggle with regional accents, word choices, or phrasing that deviate from its training data. This linguistic limitation has been noted in other studies as a common barrier to achieving high accuracy in AI-based assessments, especially in multilingual or dialect-rich contexts [24]. Addressing these linguistic challenges through improved training datasets or natural language processing models that account for regional diversity may enhance the performance of AI systems in future assessments.

Despite these challenges, the strong agreement between the 4A program and human experts demonstrates that AI has the potential to be integrated effectively into educational assessments. As the use of AI in education continues to grow, ensuring that these systems can reliably match human judgment is crucial. The findings from this study align with previous research suggesting that with proper training and refinement, AI systems can achieve high levels of agreement with human assessors, thereby offering scalable and efficient alternatives to traditional assessment methods [2].

The second objective of this study was to evaluate the accuracy and precision of the 4A program in comparison to human experts. The results revealed a non-significant difference in performance between the AI system and the human expert, with a McNemar test (χ^2^ = 0.4, *p* = 0.527). This indicates that the 4A program’s ability to accurately and precisely assess nursing students’ cognitive skills is comparable to that of human experts. This finding is consistent with previous research that highlights the role of structured testing in reinforcing cognitive processes and improving learning outcomes, a phenomenon widely recognized as the testing effect [21,22]. Comparable accuracy and precision suggest that the 4A program can serve as a reliable alternative to human evaluation, particularly in educational settings where consistency and scalability are essential. The program’s design, which incorporates predefined algorithms for assessing responses, ensures a high level of standardization, reducing the potential for human bias. These findings align with previous research demonstrating the feasibility of AI systems in replicating human performance in structured tasks, such as educational assessments and cognitive evaluations [15].

Although the 4A program showed similar performance to human experts, it is essential to consider factors that could influence its evaluation outcomes. For example, the AI’s reliance on pre-programmed algorithms may limit its ability to interpret contextual nuances or adapt to linguistic diversity, particularly in cases where participants’ primary language or phrasing deviates from the system’s training data. These limitations highlight the importance of continued refinement in AI training models to ensure that systems like the 4A program remain robust across diverse populations and contexts. Despite these challenges, the results support the potential of the 4A program as a scalable and consistent tool for cognitive skill assessment. Further research should explore its application in larger and more diverse samples to validate these findings and identify areas for improvement, particularly in handling variability in language and context.

The third objective of this study was to validate the effectiveness of the 4A program by comparing its assessment outcomes with the results of the national nursing examinations. The external validation phase revealed that higher scores on the 4A program significantly predicted success in the national exams, with an odds ratio of 1.124 (*p* = 0.031). This finding supports the predictive validity of the 4A program and underscores its potential as a valuable tool in educational settings. The positive correlation between the AI program scores and national exam outcomes indicates that the 4A program is capable of providing meaningful insights into students’ readiness for high-stakes certification exams. This is particularly important in the context of nursing education, where accurate assessment of cognitive skills is crucial for ensuring that students are prepared to deliver safe and effective patient care. The ability of the AI program to predict exam success suggests that it can be effectively integrated into the broader assessment framework, complementing traditional methods and providing additional data points for educators to consider when evaluating student performance [15].

Furthermore, the feedback from participants demonstrated the strong approval of the 4A program, particularly in its ability to engage users, reflect cognitive skills, and provide relevant and clear assessments. The high ratings for scenario-based learning and the development of analytical skills suggest that the program effectively supports critical thinking. Areas for improvement include further refinement of voice-recorded response features and addressing any challenges participants face in adapting to non-traditional assessment formats. The findings of this study align with the growing body of research supporting the integration of educational technology in healthcare training. The AI-assisted answer assessment (4A) program exemplifies how AI-powered tools can enhance traditional cognitive assessments, ensuring reliability, accuracy, and external validity. Future research should explore the broader applications of AI-driven assessments within educational technology to optimize learning outcomes in nursing education. These insights provide valuable guidance for enhancing the program’s usability and expanding its application in broader educational contexts.

### Limitations

Several methodological limitations should be noted when interpreting this study. Firstly, the study was conducted in a specific nursing education institution and with only fourth-year nursing students, which may limit the generalizability of the findings to other settings and other student groups. Secondly, the duration of the intervention and the follow-up period for assessing long-term effects were relatively short. Future research could explore the sustained impact of the 4A program over an extended period. Thirdly, the study focused on cognitive skills assessment and did not examine other aspects, such as student satisfaction or engagement with the learning program. Lastly, the limitation of this study is the reliance on the ChatGPT-4 API for NLP-based text analysis. While this model demonstrated robust accuracy in processing nursing students’ audio transcripts, alternative NLP models such as DeepSeek, GPT-3.5, BERT, or healthcare-specific AI models might yield different results due to variations in training data, contextual understanding, and domain adaptation. Future studies could explore comparative analyses of multiple NLP models to assess their relative performance in cognitive skill evaluation within nursing education.

## 5. Conclusions

This study contributes knowledge to nursing education by demonstrating the feasibility and effectiveness of AI-assisted cognitive skill assessments using the 4A program. By integrating AI-driven speech-to-text conversion and NLP-based evaluation, this approach enhances assessment efficiency and provides timely feedback, reducing the workload for educators while ensuring a standardized evaluation process. The findings suggest that AI-powered tools can complement traditional assessment methods, particularly in evaluating complex cognitive skills such as clinical reasoning and decision-making. Future research should explore the integration of alternative NLP models, assess the long-term impact of AI-assisted assessments on student learning outcomes, and evaluate their scalability across diverse nursing curricula. Additionally, investigating students’ and educators’ perceptions of AI-driven assessments could provide insights into optimizing user engagement and acceptance. For nursing educators, we recommend adopting AI-assisted assessments as a supplementary tool to support competency-based learning. Educators should also receive training in AI literacy to interpret AI-generated results effectively and ensure ethical implementation in assessment practices. As AI continues to evolve, interdisciplinary collaboration between nursing educators and AI developers will be crucial in refining these tools to align with nursing education standards and best practices.

## Figures and Tables

**Table 1:** Number and percentage of the participants in each round.

Variable	Category	Round 1 (n = 170)	Round 2 (n = 138)
Gender	Male	9 (5.29)	6 (4.35)
	Female	161 (94.71)	132 (95.65)
Hometown	North	139 (81.77)	113 (81.88)
	Central	20 (11.76)	11 (7.97)
	South	5 (2.94)	3 (2.17)
	West	0 (0.00)	1 (0.73)
	East	6 (3.53)	10 (7.25)
Primary language used	Central	95 (55.88)	91 (65.94)
	North-east	0 (0.00)	2 (1.45)
	North	68 (40.00)	45 (32.61)
	South	7 (4.12)	0 (0.00)
Main device used for testing	PC	11 (6.47)	4 (2.90)
	Notebook	32 (18.83)	32 (23.19)
	iPad	101 (59.41)	89 (64.49)
	Smartphone	26 (15.29)	13 (9.42)
GPA	Mean (SD)	3.08 (1.28)	3.14 (1.32)
AI Score	Non-pass	117 (68.82)	96 (69.57)
	Pass	53 (31.18)	42 (30.43)
National examination	Non-pass		16 (11.59)
	Pass		122 (88.41)

**Table 2:** Intraclass correlation coefficient (ICC) for agreement (n = 52).

Comparison	ICC	95%CI	*p*-Value
Human expert 1 vs. human expert 2	0.971	0.832–0.995	0.001
AI vs. human expert	0.886	0.334–0.980	0.009

**Table 3:** Comparison of accuracy, precision, and McNemar’s test between the 4A program and human experts (n = 52).

	TP	FP	FN	TN	Accuracy	Precision	McNemar Test	*p*-Value
4A program	42	4	6	0	0.808	0.913	0.4	0.527
Human expert	48	0	4	0	0.923	1.000		

TP (true positives): The number of correct responses identified accurately by the program or expert. FP (false positives): The number of incorrect responses misclassified as correct. FN (false negatives): The number of correct responses misclassified as incorrect. TN (true negatives): The number of incorrect responses correctly identified as incorrectly. Accuracy: The proportion of all correct classifications (both true positives and true negatives) to the total number of classifications. Precision: The proportion of true positives out of all instances classified as positive (true positives and false positives).

**Table 4:** External validation results (n = 138).

Outcome	Predictor	Odds Ratio	*p*-Value
National Nursing Examination	4A program score	1.124	0.031

**Table 5:** Participants feedback on 4A program.

Item	Range	Round 1 (n = 170)	Round 1 (n = 138)	Total (n = 308)
1. Online cognitive skill measurement is interesting	1–5	4.16 (0.83)	4.44 (0.67)	4.29 (0.78)
2. Online cognitive skill assessment reflects your cognitive skills well	1–5	4.27 (0.86)	4.45 (0.70)	4.35 (0.79)
3. The questions in the online cognitive skill assessment are clear	1–5	4.06 (0.87)	4.30 (0.85)	4.17 (0.87)
4. The number of bases used in the test is appropriate and covers the objectives to be assessed	1–5	4.27 (0.83)	4.40 (0.73)	4.33 (0.79)
5. The given scenarios reflect/stimulate cognitive skills	1–5	4.39 (0.79)	4.62 (0.58)	4.49 (0.71)
6. The steps in conducting the online cognitive skills measurement are appropriate	1–5	4.14 (0.91)	4.31 (0.79)	4.22 (0.86)
7. The time spent on each base is appropriate	1–5	4.43 (0.81)	4.20 (0.96)	4.32 (0.88)
8. After completing the cognitive skill assessment, you have learned to think analytically and make decisions better	1–5	4.39 (0.76)	4.58 (0.58)	4.48 (0.69)
9. You think that this type of online assessment can be applied to competency assessment in other subjects	1–5	4.29 (0.87)	4.41 (0.83)	4.34 (0.85)
10. Voice-recorded responses in cognitive skill assessment can measure cognitive skills better than multiple-choice, even though the questions are in the same direction	1–5	4.03 (1.05)	4.19 (1.05)	4.10 (1.05)

## Data Availability

The corresponding author can provide the datasets created and analyzed for this work on reasonable request.

## Public Involvement Statement

No public involvement in any aspect of this research.

## Guidelines and Standards Statement

This manuscript was drafted against the STARD 2015 (Standards for Reporting Diagnostic Accuracy Studies) guidelines for diagnostic accuracy research [25].
